# A method for detecting and correcting feature misidentification on expression microarrays

**DOI:** 10.1186/1471-2164-5-64

**Published:** 2004-09-09

**Authors:** I-Ping Tu, Marci Schaner, Maximilian Diehn, Branimir I Sikic, Patrick O Brown, David Botstein, Michael J Fero

**Affiliations:** 1 Functional Genomics Facility, Stanford University School of Medicine, Stanford, CA, USA; 2 Department of Biochemistry, Stanford University School of Medicine, Stanford, CA, USA; 3 Oncology Division, Department of Medicine, Stanford University School of Medicine, Stanford, CA, USA; 4 Lewis-Sigler Institute for Integrative Genomics, Princeton University, Princeton, NJ, USA; 5 Howard Hughes Medical Institute, Stanford University School of Medicine, Stanford, CA, USA; 6 Institute of Statistical Science, Academia Sinica, Taipei, Taiwan, R.O.C

## Abstract

**Background:**

Much of the microarray data published at Stanford is based on mouse and human arrays produced under controlled and monitored conditions at the Brown and Botstein laboratories and at the Stanford Functional Genomics Facility (SFGF). Nevertheless, as large datasets based on the Stanford Human array began to accumulate, a small but significant number of discrepancies were detected that required a serious attempt to track down the original source of error. Due to a controlled process environment, sufficient data was available to accurately track the entire process leading to up to the final expression data. In this paper, we describe our statistical methods to detect the inconsistencies in microarray data that arise from process errors, and discuss our technique to locate and fix these errors.

**Results:**

To date, the Brown and Botstein laboratories and the Stanford Functional Genomics Facility have together produced 40,000 large-scale (10–50,000 feature) cDNA microarrays. By applying the heuristic described here, we have been able to check most of these arrays for misidentified features, and have been able to confidently apply fixes to the data where needed. Out of the 265 million features checked in our database, problems were detected and corrected on 1.3 million of them.

**Conclusion:**

Process errors in any genome scale high throughput production regime can lead to subsequent errors in data analysis. We show the value of tracking multi-step high throughput operations by using this knowledge to detect and correct misidentified data on gene expression microarrays.

## Background

**Figure 1:**
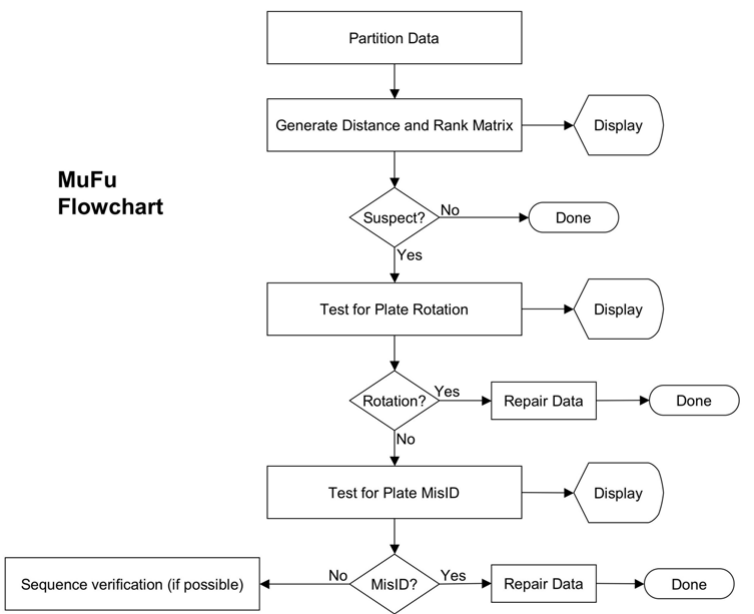
**MuFu flowchart. **Flow of the MuFu algorithm. Looping and re-partitions of the data are not shown.

Expression microarrays, with the capability to measure the mRNA expression level of tens of thousands of genes simultaneously, have found broad application in both clinical and basic research [1-7]. With the generation of large data sets from microarray experiments, statistical methods are needed to extract useful information. Many methods have had specific implementations written for the analysis of gene expression data, such as various forms of clustering, self ordered maps, singular value decomposition and significance analysis [8-13]. However, these methods all rely on the assumption that there are no gross process errors in the original data. Previous analyses of systematic errors in microarray data have focused on problems at the level of sample preparation, labelling, or hybridization. This report focuses on steps in the microarray production process prior to hybridization that may ultimately result in errors in the underlying data. Much of the microarray data published at Stanford is based on mouse and human arrays produced under controlled and monitored conditions at the Brown and Botstein laboratories and at the Stanford Functional Genomics Facility (SFGF). Nevertheless, as large datasets based on the Stanford Human array began to accumulate, a small but significant number of discrepancies were detected that required a serious attempt to track down the original source of error. Due to a controlled process environment, sufficient data was available to accurately track the entire process leading to up to the final expression data. In this paper, we describe our statistical methods to detect the inconsistencies in microarray data that arise from process errors, and discuss our technique to locate and fix these errors. We are able to fix those errors that originate at the level of any microtiter plate used during a multi-step microarray production process. The major process fail points in cDNA microarray production at Stanford are shown in Table 1. It is at these points that misidentifications can occur. Other types of processes resulting in expression data will have their own possible fail points. Regardless of the particular process, for the sake of subsequent error checking it is important to track every instance where samples have moved from one microtiter plate to another, or from microtiter plate to microarray. Our process involves four such reallocations: From an archival 96-well block to a 96-well growth block, then to a 96-well PCR plate, then to four 384-well print plates and finally to 250 10–40 thousand element microarrays. Even in highly automated bar-coded and vision controlled systems the possibility exists that plates might become swapped, skipped, misordered, or rotated by 180 degrees during one of the process steps. Our own process is fairly well automated, but like most academic and commercial efforts our process involves hand loading of robots. For example in our case, during the transfer from 96 to 384-well format, it is possible to accidentally rotate a 96-well plate or misorder the 96 or 384-well plates. During printing a 384-well plate might be accidentally skipped, swapped or printed in the wrong orientation (rotated by 180 degrees). Even with the best engineering controls to prevent plate rotations, the potential exists for misidentified plates. Inconsistencies in our data were first detected both by visual inspection of microarray data as well as the appearance of anomalously large first components in singular value decomposition analyses of Ovarian Tumour data [6] traced to different production batches of arrays. Our algorithm was developed to detect and repair these types of errors, using the similarities in expression levels between sets of spots from different microarrays. The algorithm was used to check all of the microarray data in our database for which there was a sufficient process record. The general idea, illustrated in Figure 1, is as follows: Partition the microarray expression data from a single microarray into sets based on the various microtiter plates the samples have been in throughout their process history. For example, at Stanford we keep our process fairly simple, with a minimum of plate changes, so our spotting material can be said to have existed in either 96 well or 384-well format during its entire process history. Thus, we partition the data into sets corresponding to the 96 or 384-well plate history. Next, an expression vector for each element of the partition (each plate) based on absolute (not relative) expression levels is formed and compared to every other expression vector from every other plate on many other array batches. A rank matrix of correlation coefficients is formed which should be close to unity on the diagonal and far from unity off the diagonal. Non-unitary diagonal elements indicate problems with that plate comparison. A rank comparison of the best correlations can be made to find swapped plates. The algorithm can be repeated assuming a rotated plate to check to see if the discrepancy can be attributed to a plate rotation. It should be noted that in cases where no process error is indicated, the method can still indicate the presence of problem plates, print batches or PCR rounds that should be flagged for particular attention in downstream analyses. Our algorithm (named MuFu for "MixupFixup") is for arrays produced in the Brown and Botstein laboratories and the Stanford Functional Genomics Facility. However, the ideas are general and can be applied to many other types of high throughput operations. In most of the research studies using our human microarray, a common reference [14] is compared against a tumour or tissue specimen. The common reference is usually labelled with the Cy3 dye, pseudo-colour green in most visualizations of the data, while the sample specimens are labelled with Cy5 dye, pseudo-colour red. Subtle corrections due to background subtraction issues and normalization are not important for this analysis. Because a common reference is used in a large number of hybridization experiments at Stanford, all of the Cy3 intensities (or Cy5 in some cases) across various kinds of experiments are comparable. We measure the similarities of two sets of spots by taking the correlations between the common reference intensities of these two sets of spots. For those experiments that do not use common reference, the comparison is made using experiments with similar samples in either the Cy3 or Cy5 channel.

## Results

### Finding misidentified data

**Figure 2:**
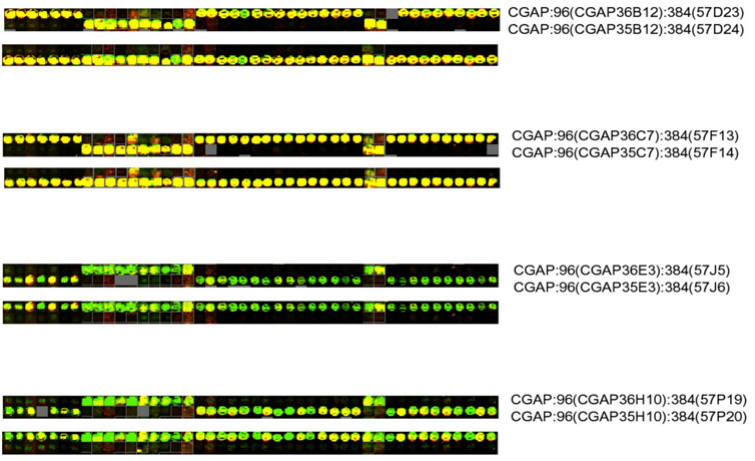
**Ovarian tumour data. **Visual inspection of the anomalous spots from the Ovarian tumor data before and after applying MuFu. In each case the top row shows that the feature alignment is inconsistent with the contents of the plate. In the bottom row features group together as expected.

This example is from the analysis of experiments from an ovarian tumor dataset[6] that first led us to develop MuFu. Here, it was noticed that similar tissues were not clustering across batch boundaries as expected. Also, an anomalously large first component in a singular value decomposition analysis[9] pointed to problems at the array batch level. After some detective work, visual inspection uncovered anomalies in certain print plates as seen in Figure 2. We were able to isolate the problem to distinct sets of 96-well plates that had been swapped during an upstream process step, probably during the transfer from 96-well plates to 384-well plates. Not wishing to go through this sort of process again and again, MuFu was developed to more succinctly recapitulate this finding and apply it to all data.

With MuFu, a check using a 384-well plate partition of the data shows a discrepancy but no obvious plate rotation or misidentification. We then repartition and repeat our tests at the 96-well plate level. The results are shown in Table 2a where we show a 12 way pair wise comparison. The first four comparisons are for batches whose print plates are made from the first PCR round. The middle four compare the first PCR round to the second, and the last four compare batches from the second PCR round. Mismatches are evident in the middle set of comparisons. From Table 2b we see that the distance matrix identifies a match between plate *h *and plate *i *and plate *n *and plate *o *for all four comparisons of the first PCR round to the second.

**Table 2:** Test for 96 well plate handling error. a) In this table we see that the middle bank of comparisons indicates a discrepancy between data from the first PCR run and the second, at the 96-well plate level. b) A check of the distance matrix results show that the swapped plates are *h *and *i *in one case, and *n *and *o *in the other. Bold indicates P-value = 0.3, italic indicates P-values between 1.0E-03 and 1.0E-04, while regular font indicates P-values < 1.0E-04.

a)	R_{m, m}											
	
	PCR Round 1 vs 1				PCR Round 1 vs 2				PCR Round 2 vs 2			
Plates	A1 v A2	A2 v A3	A3 v A4	A4 v A5	A2 v B1	A3 v B2	A4 v B3	A5 v B4	B1 v B2	B5 v B3	B1 v B4	B5 v B4

{a, a}	1	1	1	1	1	1	1	2	1	1	2	1
{b, b}	1	1	1	1	1	1	1	1	7	1	3	1
{c, c}	1	1	1	1	1	1	1	1	1	1	1	1
{d, d}	2	1	1	1	1	1	5	9	1	1	1	1
{e, e}	1	1	1	1	1	1	1	2	1	1	2	1
{f, f}	1	1	1	1	1	1	1	1	1	1	1	1
{g, g}	3	1	1	1	1	1	1	1	1	2	1	1
{h, h}	1	1	1	1	**343**	**322**	**408**	**294**	1	1	1	1
{i, i}	2	1	1	1	**421**	**290**	**402**	**359**	1	1	1	1
{j, j}	1	1	1	1	2	1	1	1	2	1	4	1
{k, k}	1	1	1	1	2	1	1	1	1	1	1	1
{l, l}	1	1	1	1	1	2	1	1	1	1	1	2
{m, m}	1	1	1	1	1	1	1	1	1	1	1	1
{n, n}	1	1	1	1	**255**	**141**	**20**	**167**	1	1	1	1
{o, o}	1	1	1	1	**330**	**248**	**357**	**277**	3	1	5	6
{p, p}	1	1	1	1	1	3	1	1	3	2	1	1
{q, q}	1	1	1	1	4	3	3	2	1	1	1	1
{r, r}	1	1	1	1	1	1	1	5	1	1	5	1
{s, s}	3	1	1	1	1	1	1	1	1	1	1	1
{t, t}	1	1	1	1	1	1	1	1	1	1	1	1
{u, u}	1	1	1	1	1	1	1	1	1	1	1	1
{v, v}	1	1	1	1	1	1	7	1	1	1	1	1
{w, w}	2	1	1	1	1	16	1	2	1	1	1	1
												
												
b)	A2 v B1			A3 v B2			A4 v B3			A5 v B4		
	
	D_{mm}		D_{mn}	D_{mm}		D_{mn}	D_{mm}		D_{mn}	D_{mm}		D_{mn}

MEAN	0.33		0.97	0.34		0.89	0.44		0.98	0.37		0.96
SD	0.2		0.11	0.2		0.13	0.21		0.11	0.21		0.12
{h, h}	0.99			1.02			*1.07*			*1*		
{i, i}	1.09			1			*1.07*			1.06	
{h, i}			0.22			0.09			0.33			0.31
{i, h}			0.28			0.07			0.11			0.14
{n, n}	0.98			*0.91*			**0.85**			*0.92*		
{o, o}	1.01			1.02			*1.07*			1.02		
{n, o}			0.1			*0.51*			0.45			0.52
{o, n}			0.04			0.17			0.11			0.05

**Figure 3:**
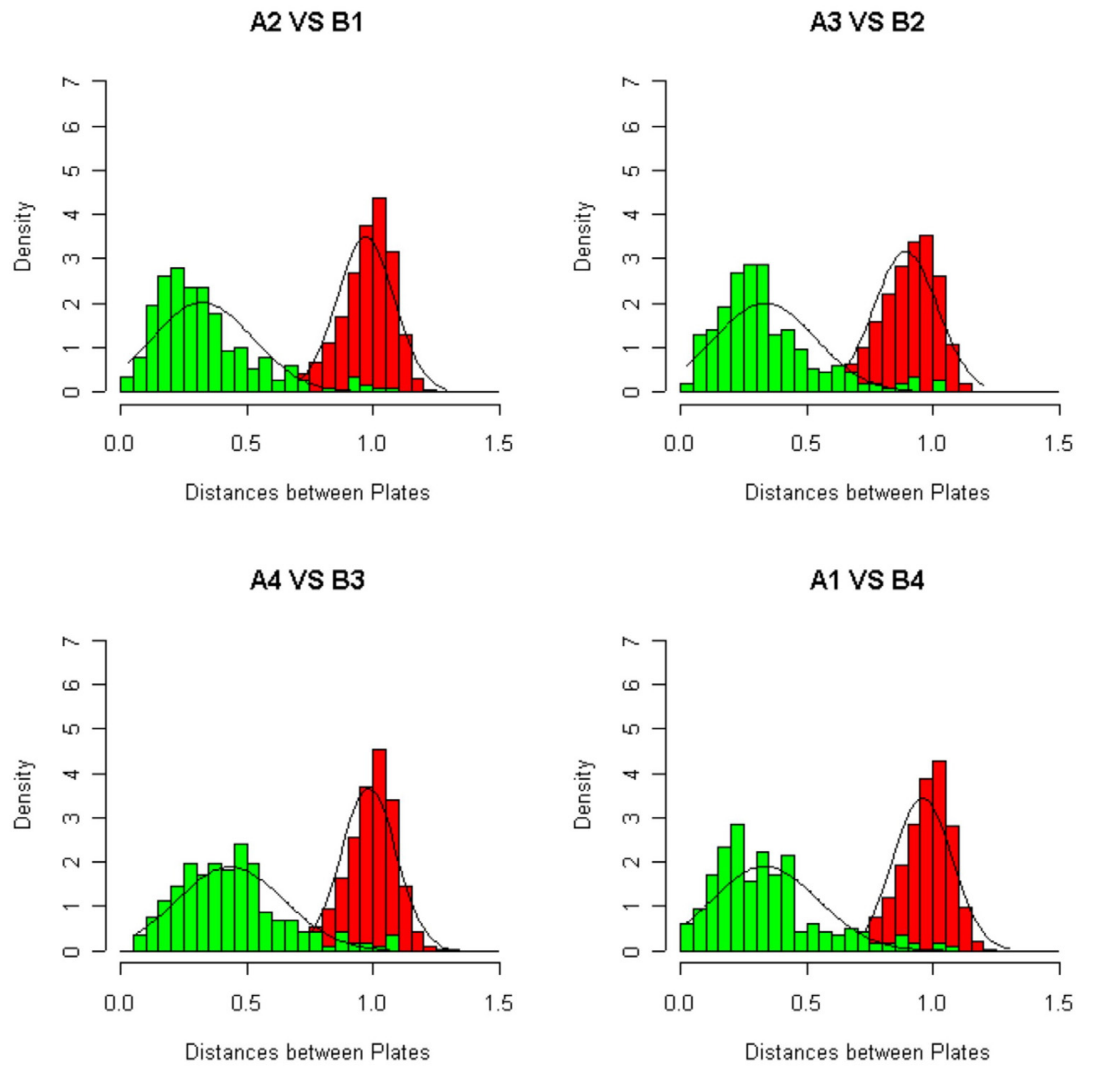
**Match, mismatch distance distributions. **Good separation between match and mismatch distance distributions at the 96-well plate level lends confidence to our ability to discriminate between chance matches and actual matches. The green bars refer to the distance distributions of matched plates and the red bars for mis-matched plates.

**Figure 4:**
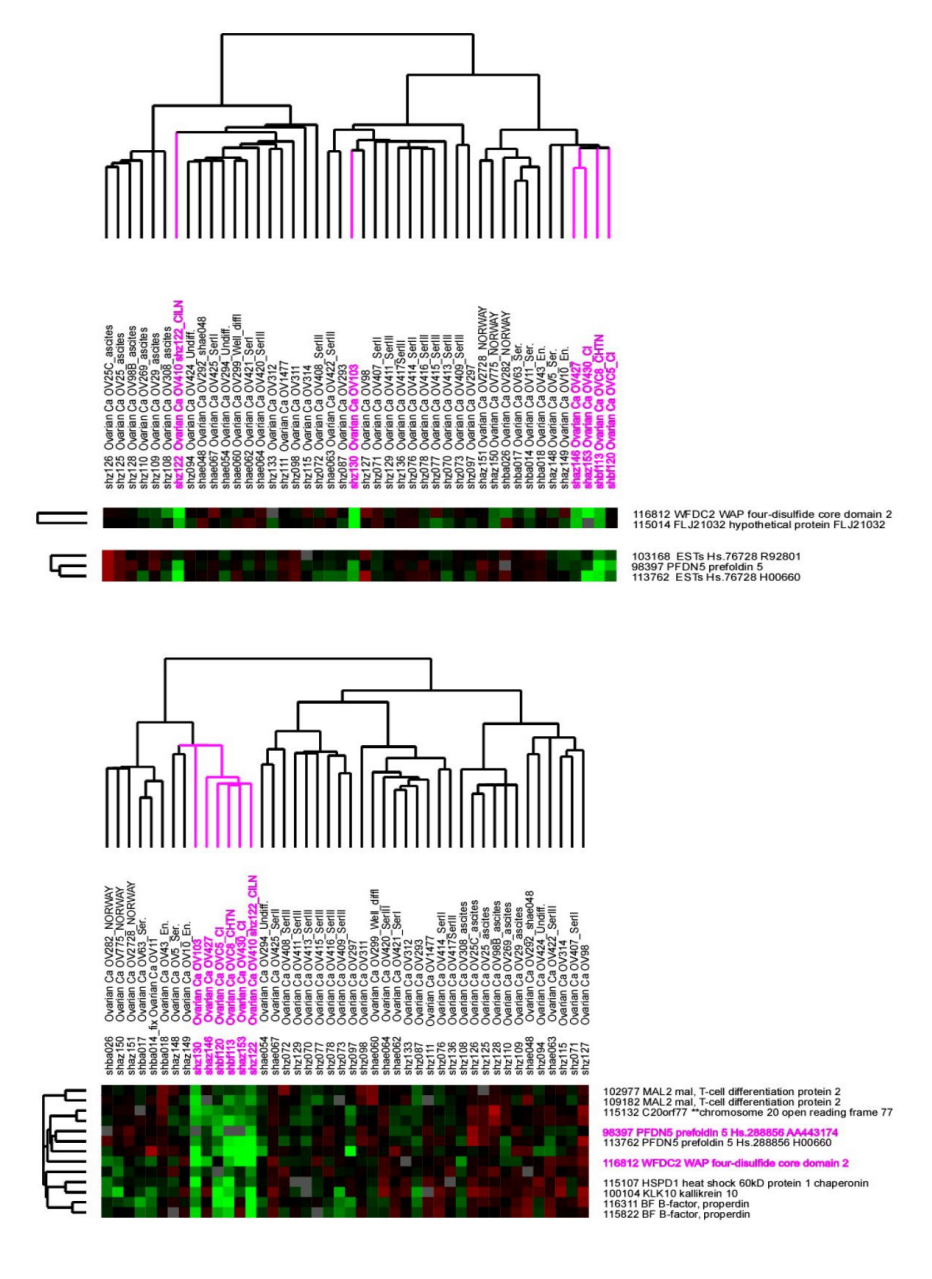
**Ovarian tumour clusters. **In Ovarian tumor data it was noticed that similar experiments were not clustering as expected (upper cluster diagram). Using MuFu we were able to isolate the problem to a distinct set of 96-well plates that had been swapped during an upstream process step, probably during the transfer from 96-well plates to 384-well plates. The samples cluster together as expected after correction (lower cluster diagram).

In all four comparisons, the two distributions resolve themselves well, as can be seen in Figure 3, leading to the conclusion that *h *and *i *are swapped, and *n *and *o *are swapped, most likely in the plates from the first PCR round. The ambiguity is broken by sequencing a small sampling of wells, which confirms that the misidentifications are in the print plates from the first round of PCR, and not the second. In Figure 4 we show the effect on the Ovarian data after the correction has been applied. Also, in Figure 2 we show how, via visual inspection of the actual spots on an array, one can verify that the fix has properly reorganized the data. The figure shows spots from the four different affected 96-well plates before and after the fix is applied.

### Finding a 384-well plate rotation

**Figure 5:**
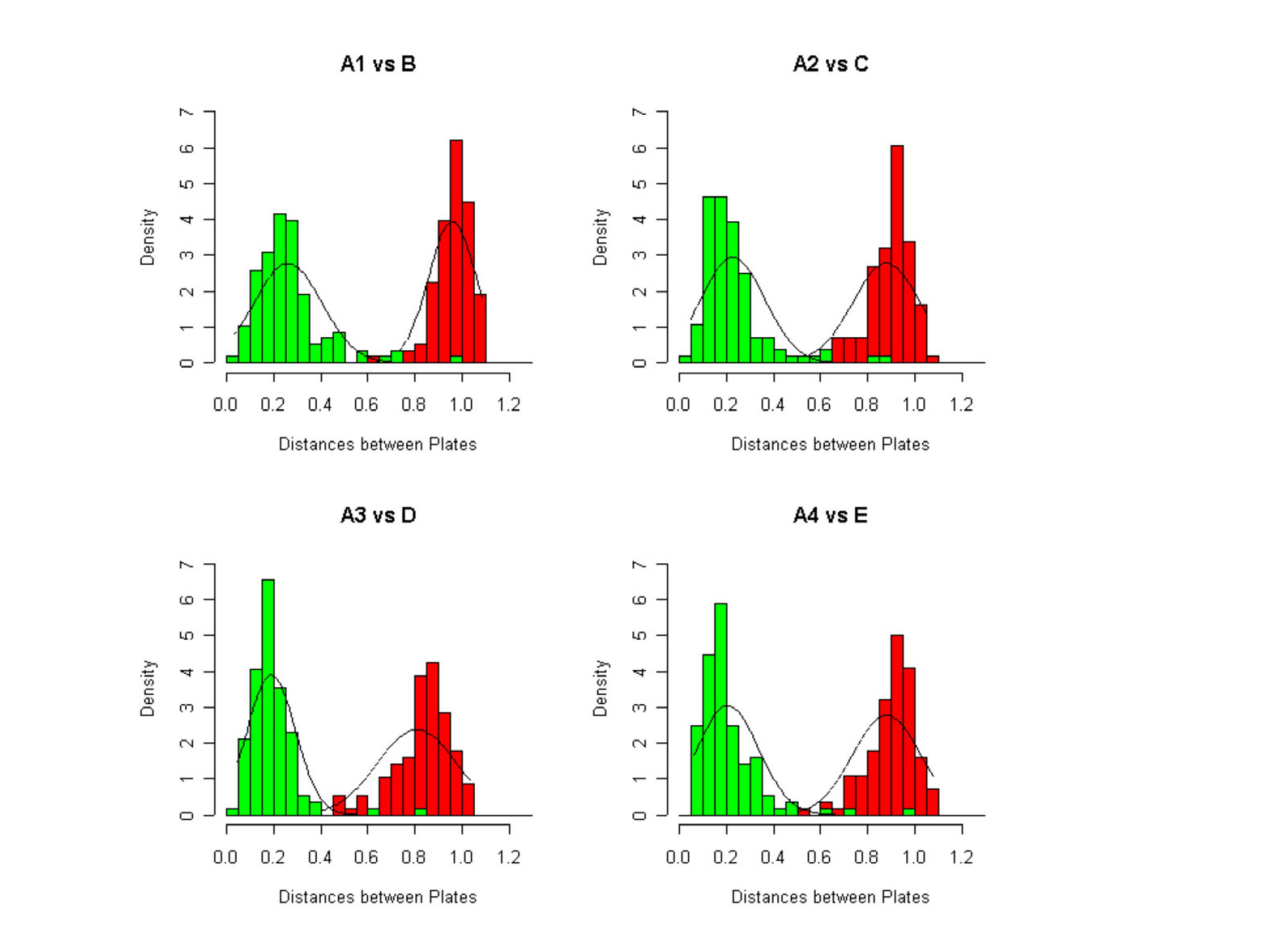
**Plate rotation distance comparison. **Histogram of the distance comparisons for the plate rotated and not rotated cases. The distributions are well separated, where the green bars refer to the distance distributions for non-rotated plates and the red bars for rotated plates.

We compare four arrays A1, A2, A3 and A4 from a particular print production batch A to four arrays B, C, D and E from four other distinct print production batches. The total number of 384 well microtiter plates in print batch A is 113. The results of the plate rotation test are shown in Table 3a. Here we see plate *p*, identified in both the rank and rotated-rank matrices across print batches is a clear candidate for a plate rotation. The data from the distance matrix comparisons is shown in Table 3b. In Figure 5 we show the distributions of the diagonal elements of the distance matrix and the rotated-distance matrix. These distributions are well resolved and allow us to easily distinguish data due to a plate rotation from noisy data.

**Table 3:** Test for rotated plates. a) Plate rotation results from the rank matrices R and R' . The flagged comparisons indicate a plate rotation for plate *p*. b) Plate rotation distance matrix comparison. The data meet the criteria for a plate rotation.

a)	A1 v B		A2 v C		A3 v D		A4 v E	
Plates	R_{mm}	R'_{mm}	R_{mm}	R'_{mm}	R_{mm}	R'_{mm}	R_{mm}	R'_{mm}

{i, i}	1	102	*1*	95	*1*	82	*1*	93
{j, j}	1	18	*1*	102	*1*	16	*1*	64
{k, k}	1	52	*1*	99	*1*	102	*1*	41
{l, l}	6	108	*3*	35	**9**	76	*1*	22
{m, m}	1	51	*1*	79	*1*	85	*1*	68
{n, n}	1	14	*1*	35	*1*	41	*1*	93
{o, o}	1	92	*1*	92	*1*	102	*1*	76
{p, p}	**78**	**1**	**48**	**1**	**46**	**1**	**65**	**1**
{q, q}	1	117	*1*	96	*1*	87	*1*	111
{r, r}	1	118	*1*	110	*1*	115	*1*	112
{s, s}	1	116	*1*	108	*1*	109	*1*	113
{t, t}	1	69	*1*	93	*3*	20	*1*	103
{u, u}	1	116	*1*	103	*1*	72	*1*	84
{v, v}	1	83	*1*	104	*1*	85	*1*	64
								
								
b)	A1 v B		A2 v C		A3 v D		A4 v E	

	D_{mm}	D'_{mm}	D_{mm}	D'_{mm}	D_{mm}	D'_{mm}	D_{mm}	D'_{mm}

MEAN	0.26	0.96	0.23	0.88	0.19	0.81	0.21	0.88
SD	0.14	0.1	0.14	0.14	0.1	0.17	0.13	0.14
{pp}	0.95		0.84		0.85		0.97	
{pp}		0.21		0.14		0.23		0.24
P-value	4.10E-07	3.20E-14	6.60E-06	6.30E-08	2.10E-11	3.20E-04	2.50E-09	2.40E-06

### Finding a misidentified 384-well plate

**Figure 6:**
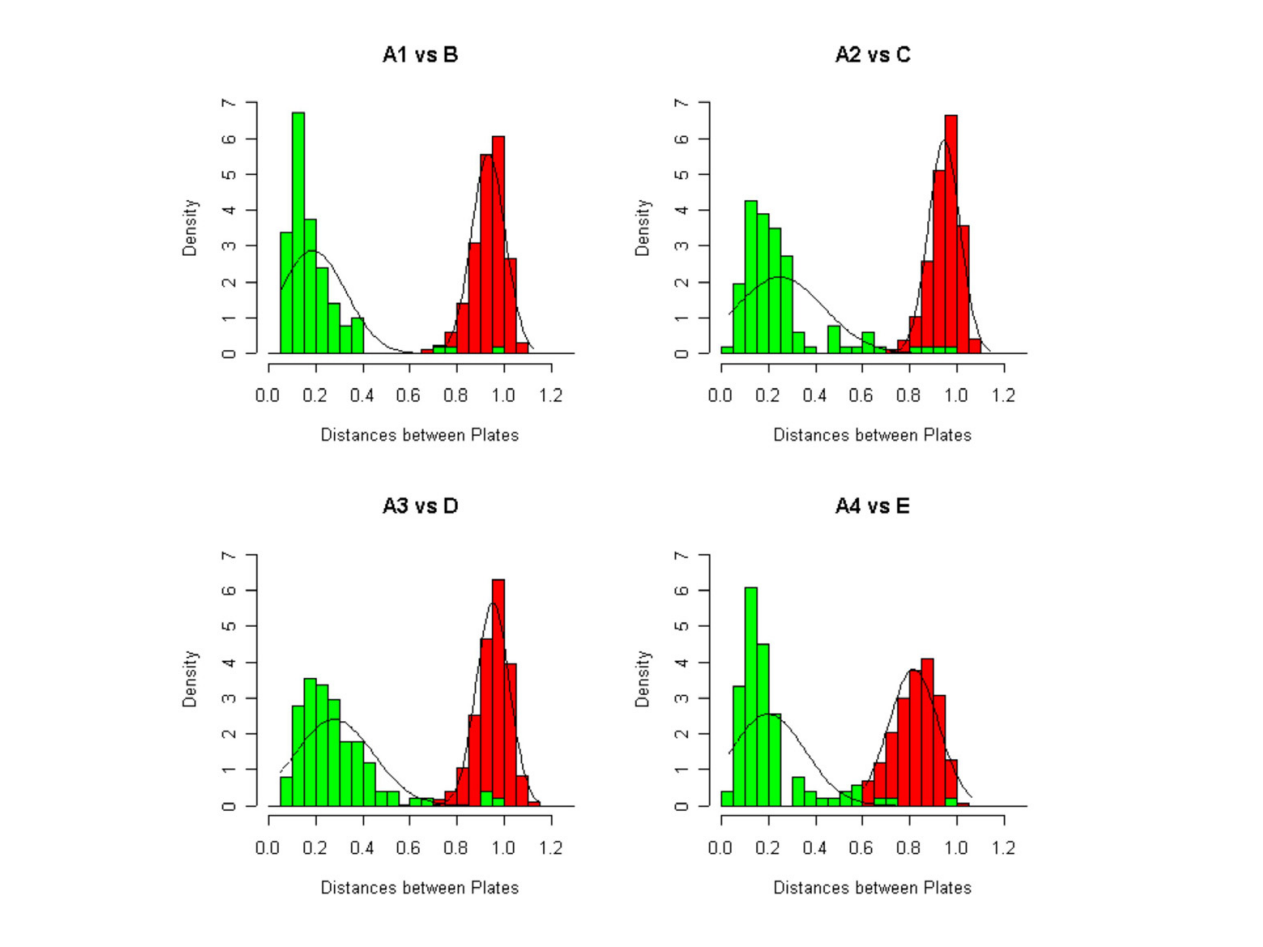
**Misidentification distance comparison. **Misidentified plate distance histogram showing good separation between the match and mismatch distance distributions. The green bars refer to the distance distributions of matched plates and the red bars for mis-matched plates.

In this example we compare four arrays A1, A2, A3 and A4 from a particular print batch A to four other arrays B, C, D and E from four other distinct print batches. As seen in Table 4a no plate is found to be a candidate for plate rotation, however we do find that plate *q *has a poor self-match comparison. Indeed, when the distance matrix is examined, we see the plate matches instead plate *r *across the four array comparisons. We check the three criteria for plate misidentification and summarize these data in Table 4b. In Figure 6 we show the distributions of the match and mismatch distributions. These distributions are quite distinct and give us confidence that we can distinguish the proper match from an accidental match.

**Table 4:** Test for swapped plates. a) Plate *r *is seen here to have a problem, but from the table we see that it is most certainly not a plate rotation. b) A check of the distance matrix shows the *{r, q} *comparison to be quite good and satisfies the criteria for a match, indicating that plates *r *and *q *have been accidentally swapped.

a)	A1 v B		A2 v C		A3 v D		A4 v E	
Plates	R_{mm}	R'_{mm}	R_{mm}	R'_{mm}	R_{mm}	R'_{mm}	R_{mm}	R'_{mm}

{i, i}	1	82	1	48	1	107	1	76
{j, j}	1	45	1	87	1	87	1	89
{k, k}	1	18	1	45	1	63	1	15
{l, l}	1	85	1	106	1	103	1	93
{m, m}	1	30	1	49	1	53	1	37
{n, n}	1	115	1	113	1	37	1	102
{o, o}	1	24	1	64	1	82	1	100
{p, p}	1	110	1	102	1	104	1	93
{q, q}	1	118	1	111	1	110	1	91
{r, r}	**77**	7	**48**	12	**51**	22	**16**	23
{s, s}	1	55	1	44	1	90	1	88
{t, t}	1	115	1	101	1	110	1	99
{u, u}	1	118	1	93	1	74	1	95
{v, v}	1	15	1	13	1	29	1	71
								
								
b)	A1 v B		A2 v C		A3 v D		A4 v E	

	D_{mm}	D_{mn}	D_{mm}	D_{mn}	D_{mm}	D_{mn}	D_{mm}	D_{mn}

MEAN	0.19	0.93	0.25	0.94	0.28	0.95	0.2	0.81
SD	0.14	0.07	0.19	0.07	0.17	0.07	0.16	0.1
{rq}		0.3		0.26		0.25		0.34
{rr}	0.97		0.95		0.98		0.72	
P-value	1.30E-08	0	1.10E-04	0	1.90E-05	0	5.80E-04	1.30E-06

## Discussion

Out of the 265 million features checked in our database using MuFu, problems were detected and corrected on 1.3 million of them. That we were able to find and correct both previously known and unknown problems gives us confidence in the algorithm. That so few problems existed overall (0.5%) reassures us as to the robustness of our process in general.

Microarray data, by its sheer volume, presents interesting laboratory information management challenges. The data arrive at the investigators desk after a significant number of steps. We have found that the better you track production, quality control, and experimental steps, the better chance you have of uncovering the reasons behind discrepancies that may appear in the data. Often, statistical analyses look only at the data presented as final expression values or ratios, without taking into account relevant quality control data. In our effort to understand our errors and the source of large systematic discrepancies in our data we have found the MuFu algorithm a useful test of certain classes of process errors, and as a check for general problems with specific process steps or microtiter plates. We use MuFu to find, verify and fix problems that can be attributed to an error in plate processing. We also flag plates for which we can find no specific problem but we see yield inconsistent results. These may be plates that, at some point in the process, had a problem (cross contamination, a PCR problem) that was not detected while the process step was being carried out. The fact that we can test the data, using our standard quality control hybridizations, for these types of quality issues is reassuring and has helped us gain confidence in our data.

Obviously, there are many other classes of error that creep into microarray data. Aside from the gross process errors that are amenable to detection, as we have described here, there is also a large class of more subtle systematic errors that can contribute to the overall systematic error on the expression ratio measurement. Isolating the source of these individual errors is sometimes quite difficult. Properties of the microarray feature such as spot size, shape and uniformity can contribute, but the majority of systematic errors are introduced at the time the experiment is performed. Slide post processing, RNA quality, labelling, hybridization and washing all lend the possibility for introducing systematic errors. Improvements in protocols and hybridization apparatus have helped reduce these errors and should continue to do so in the future. As these sources of error are identified and eliminated, expression microarrays will continue to provide progressively more sensitive measures of gene expression.

## Conclusions

Process errors in any genome scale high throughput production regime can lead to subsequent errors in data analysis. We have shown the value of tracking multi-step high throughput operations by using this knowledge to detect and correct misidentified data on gene expression microarrays. We generalized our procedure using a simple heuristic, which found and fixed several problems with the proper assignment of gene identifier with physical microarray feature. We found thirteen print runs (9K arrays) that had four plates mistracked, six print runs with single 384 plate rotations, and one instance of a plate rotation at the 96 well plate level. One skipped plate was detected, as well as one plate printed twice. Out of the 265 million features checked in our database using MuFu, problems were detected and corrected on 1.3 million of them. That we were able to find and correct both previously known and unknown problems gives us confidence in the algorithm. That so few problems existed overall (0.5%) reassures us as to the robustness of our process in general. A list of corrected arrays can be found at .

## Methods

We follow the simple heuristic outlined here. The flowchart for the program is shown in Figure 1. In the figure we do not include additional loops needed to repartition the data in different ways for different scenarios.

### Data partitioning

1. Begin by partitioning the gene expression data on an array into subsets according to plate. Other partitions can also be made but the plate level partition is the most useful for our purposes. Let *A*_*ij *_be the intensity measurements of array *A*, subset *i *and gene index *j*. The measurements are usually of the channel (Cy3 or Cy5) used as a common reference. For example, if we partition the data by the 384-well plate each feature once occupied at some point in its process history, *A *is the array id, *i *is the plate id, and *j *is the well index between 1–384. Let *A*_*i *_be the vector (*A*_*i*1_,...,*A*_*i*384_). In our vernacular this is the 384-well plate expression vector for plate *i*. Let *B*_*ij *_and*B*_*i *_be the similar definitions for array *B*. We also reverse the data vectors from array *A *and label it *A*_*i*_' . In our notation, *A*_*i*_' = (*A*_*i*384_,...,*A*_*i*1_) is the expression vector for a plate rotated by 180°.

2. Generate a distance matrix {*D*_*mn*_, 1 ≤ *m, n *≤ *N*} in which each element *D*_*mn *_= 1 - *corr*(*A*_*m*_, *B*_*n*_). For the 384-well plate example, *A*_*m *_is the 384-well expression vector for plate *m *on array *A*, *B*_*n *_is the 384-well expression vector for plate *n *on array *B*, and *D*_*mn *_is the distance between the two vectors in this 384 dimensional space. We also generate the corresponding rotated-distance matrix {*D'*_*mn*_, 1 ≤ *m, n *≤ *N*} for the plate rotation case, in which each element *D'*_*mn *_= 1 - *corr*(*A'*_*m*_, *B*_*n*_). We tried several correlation functions including Euclidean and Pearson but found the Pearson to be best suited to this task.

3. Generate a rank matrix {*R*_*mn*_, 1 ≤ *m, n *≤ *N*} by converting the distances to ranks such that the row *m *in the rank matrix is the order statistic of the row *m *of the distance matrix. We also generate the corresponding rotated-rank matrix {*R'*_*mn*_} for {*D'*_*mn*_}. Ideally, for the case where we are comparing identical subsets from two different arrays we expect the diagonal elements of the rank matrix to all be equal to one, which means that each subset of genes from the first array matches its corresponding subset in the second array the best. In general, due to the statistical variation in array data, the diagonal elements are not all equal to one, even if there are no misidentification errors. The examples show that this does not hinder us from making a clear distinction between real problems in the data and statistical fluctuations.

### Identification of rotated plates

A plate rotation may have an affect on a single microarray batch if it occurs during array printing or may persist across many print batches if it happens during a 96-well (PCR) process step. In any case, the mismatch will persist across many array comparisons. To check for plate rotations in a print batch, we compare an array sample (*A1, A2, A3, A4 *in the example) from the print batch in question to a control sample of arrays (*B, C, D, E *in the example) selected from several distinct print batches. By comparing the rank *R*, and rotated-rank *R'*, matrix assignments for comparisons across array batch boundaries we can quickly flag possible rotated plates. A visualization of this test is shown in Table 3a. In the table we have flagged the top 5% of all ranks in the rotated column and the bottom 95% in the unrotated column. If the flags agree across all comparisons, we have a strong indication that a plate rotation has occurred. If we see a flag raised in this test, but we cannot attribute it to a plate rotation, this may indicate a different class of process error. In particular, if the flag is raised for all pairwise comparisons of the batch being tested (in this case batch *A*) against all of the control batches (in this case batches *B, C, D *and *E*) in the non-rotated case, we conclude that the corresponding flagged plate or partition from batch *A *may be misidentified. Note that in the limit that the partitions of the array are all uniform in expression ratios there is a 5% probability of a spurious flag. For this reason it is better to use high quality, highly variegated control arrays for such tests. Next, to better resolve ambiguous cases and to check our rank matrix determination we use the distance rather than rank matrix. If, for example, a plate *x *is to be considered a rotated plate, the following three criteria must be met.

1. *D'*_*xx *_<*D*_*xx*_. The rotated-distance must be less than the non-rotated distance.

2. *D'*_*xx *_is close to the mean of the distribution, {*D*_*mm*_, 1 ≤ *m *≤ *N*}, and D_*xx *_is close to the mean of the distribution {*D'*_*mm*_, 1 ≤ *m *≤ *N*}.

3. *D'*_*xx *_is an outlier of the distribution {*D'*_*mm*_, 1 ≤ *m *≤ *N*}, and *D*_*xx *_is an outlier of the distribution {*D*_*mm*_, 1 ≤ *m *≤ *N*}.

The second and third criteria above are valid if the distributions of {*D'*_*mm*_, 1 ≤ *m *≤ *N*} and {*D*_*mm*_, 1 ≤ *m *≤ *N*} separate well. In Figure 5 we showed that our data support this model.

### Finding misidentified plates

If a plate has consistently poor self-match rankings and a plate rotation has been excluded as a possible source of error, this next step tests for a plate misidentification. From the rank matrix we can identify the best alternative match. We test a suspect plate *x *for a match with plate *y *with the following criteria:

1. *D*_*xy *_<*D*_*xx*_. The mismatch distance is shorter than the match distance.

2. *D*_*xy *_is close to the mean of the distribution {*D*_*mm*_, 1 ≤ *m *≤ *N*}, and *D*_*xx *_is close to the mean of the distribution {*D*_*mn*_, 1 ≤ *m *≠ *n *≤ *N*}.

3. *D*_*xx *_is an outlier of the distribution {*D*_*mm*_, 1 ≤ *m *≤ *N*}, and *D*_*xy *_is an outlier of the distribution {*D*_*mn*_, 1 ≤ *m *≠ *n *≤ *N*}.

Again, the second and third criteria above are valid if the distributions of {*D*_*mm*_, 1 ≤ *m *≤ *N*} and {*D*_*mn*_, 1 ≤ *m *≠ *n *≤ *N*} are well resolved.

## Authors' contributions

IT, MF developed the heuristic and carried out analysis and implementation.

MS brought this problem to our attention and provided test data.

MD provided advice and checked our work with an alternative technique.

All authors read and approved the final manuscript.

**Table 1:** Process steps The process fail points for cDNA based microarray production. Steps shown in italics are accessible to the testing methods outlined here. Earlier steps may require sequencing to test. The process ID is used to identify steps where the problems, if any, are found.

Process ID	Problem Type	Note
-999	Unidentified	
0.0	Source.General	persists across all arrays at clone, 96 level
0.1	Source.Contamination	
0.2	Source.MisID	
1.0	Prep.General	persists across all arrays at clone, 96 level
1.1	Prep.Contamination	
1.2	Prep.MisID	
1.2.1	Prep.MisID.OrderError	
1.2.2	Prep.MisID.Rot	
1.3	Prep.Fail	
2.0	*PCR.General*	persists for 1 PCR round at 96 level
2.1	*PCR.Contamination*	
2.2	*PCR.MisID*	
2.3	*PCR.Fail*	
2.4	*PCR.Cleanup*	
3.0	*Replate.General*	persists for 1 PCR round at 96 level
3.1	*Replate.Contamination*	
3.2	*Replate.MisID*	
3.2.1	*Replate.MisID.OrderError*	
3.2.2	*Replate.MisID.96Rot*	
3.2.3	*Replate.MisID.384Rot*	
4.0	*Print.General*	persists for 1 print run batch at 384 level
4.1	*Print.Contamination*	
4.2	*Print.MisID*	
4.2.1	*Print.MisID.OrderError*	
4.2.2	*Print.MisID.Rot*	
4.3	*Print.Fail*	dried out plate, too concentrated, too weak etc.
5.0	Scan.General	anything after array production
